# Explaining the complex impact of the Covid-19 pandemic on children with overweight and obesity: a comparative ecological analysis of parents’ perceptions in three countries

**DOI:** 10.1186/s12889-022-13351-1

**Published:** 2022-05-17

**Authors:** P. Nowicka, A. Ek, I. E. Jurca-Simina, C. Bouzas, E. Argelich, K. Nordin, S. García, M. Y. Vasquez Barquero, U. Hoffer, H. Reijs Richards, J. A. Tur, A. Chirita-Emandi, K. Eli

**Affiliations:** 1 Division of Pediatrics, Department of Clinical Science, Intervention and Technology, Karolinska Institutet, Stockholm, Sweden; 2 Department of Food Studies, Nutrition and Dietetics, University of Uppsala, Uppsala, Sweden; 3 Department of Microscopic Morphology Genetics Discipline, Center of Genomic Medicine, “Victor Babes” University of Medicine and Pharmacy Timisoara, Timisoara, Romania; 4 CIBER of Physiopathology of Obesity and Nutrition (CIBEROBN), Instituto de Salud Carlos III, Madrid, Spain; 5 Research Group on Community Nutrition and Oxidative Stress, University of the Balearic Islands, Palma de Mallorca, Spain; 6 School of Anthropology and Museum Ethnography, University of Oxford, Oxford, UK; 7 Warwick Medical School, University of Warwick, Coventry, UK

**Keywords:** Ecological system theory, Food, Physical activity, Resilience, Qualitative

## Abstract

**Background:**

The Covid-19 pandemic has changed children’s eating and physical activity behaviours. These changes have been positive for some households and negative for others, revealing health inequalities that have ramifications for childhood obesity. This study investigates the pandemic’s impact on families of children aged 2–6 years with overweight or obesity.

**Methods:**

Drawing on interviews conducted as part of a randomised controlled trial (RCT) for childhood obesity, thematic analysis was used to examine how parents of pre-schoolers perceived changes in their eating, screentime and physical activity behaviours between the first and second waves of Covid-19. Parents (*n* = 70, representing 68 families) were interviewed twice during a period of 6 months in three countries with markedly different pandemic policies – Sweden, Romania, and Spain. The analysis is informed by Bronfenbrenner’s ecological systems theory, which embeds home- and school-based influences within societal and policy contexts.

**Results:**

The findings show that, although all participants were recruited from an RCT for families of children with excess weight, they reported different responses to the pandemic’s second wave, with some children engaging in healthier eating and physical activity, and others engaging in comfort eating and a more sedentary lifestyle. Differences in children’s obesity-related behaviours were closely related to differences in parents’ practices, which were, in turn, linked to their emotional and social wellbeing. Notably, across all sites, parents’ feeding and physical activity facilitation practices, as well as their emotional and social wellbeing, were embedded in household resilience. In resilient households, where parents had secure housing and employment, they were better able to adapt to the challenges posed by the pandemic, whereas parents who experienced household insecurity found it more difficult to cope.

**Conclusions:**

As the Covid-19 pandemic is turning into a long-term public health challenge, studies that address household resilience are crucial for developing effective prevention and treatment responses to childhood obesity.

**Supplementary Information:**

The online version contains supplementary material available at 10.1186/s12889-022-13351-1.

## Introduction

In 2020, as the Covid-19 pandemic spread around the world and governments reacted with lockdowns and school closures, researchers cautioned that this global crisis could lead to the exacerbation of an existing public health challenge – childhood obesity [1–3]. The pandemic introduced a number of stressors detrimental to children at risk for obesity, namely the closure of schools and attendant reduction in physical activity, the disruption of everyday routines, and the increase in stress and uncertainty, which could affect metabolic health [1–3]. As an emerging phenomenon, the impact of the Covid-19 pandemic on childhood obesity calls for qualitative research, whose open-ended and exploratory approach allows researchers to gain a better understanding of new and understudied phenomena, identify key issues and develop questions for future research [4]. So far, the qualitative literature has focused on educators’ and parents’ perceptions. In Canada and the US, interview-based studies found that educators perceived school closures and remote education as reducing children’s access to healthy food and physical activity opportunities [5, 6]. In the US, focus group and interview-based studies with parents found that while certain eating behaviours improved during the pandemic – specifically, home-cooked meals were prepared more frequently and family meals were more common – parents reported their children snacked more, had more screentime, consumed more food and gained weight [7, 8]. Interestingly, while some parents reported their children engaged in less physical activity due to school closure and reduced access to sporting activities and outdoor spaces, others reported their children had increased access to outdoor spaces, which resulted in increased physical activity [8]. While limited to North America, these qualitative studies have shown that the Covid-19 pandemic and responses to it have changed children’s eating and physical activity, albeit in sometimes-ambivalent ways. Deciphering this ambivalence – that is, the co-existence of positive and negative behaviour changes, and the divergent effects of the pandemic on different families – is at the core of this study.

To understand why the Covid-19 pandemic has had a complex impact on children’s eating and physical activity, we frame this study through Bronfenbrenner’s ecological systems theory [9]. According to this theory, a child develops through the interaction of multiple systems at the micro, meso and macro levels. These systems range from the nuclear family (micro), through the school and local community (meso), to national policy (macro) [9]. Bronfenbrenner’s ecological systems theory has been used to explain the complex environmental and developmental interactions that give rise to childhood obesity, embedding home- and school-based influences on eating, activity, and self-esteem within wider societal and policy contexts [10, 11]. Through an ecological systems lens, children’s eating, screen time and physical activity behaviour changes during the Covid-19 pandemic should be viewed as multifactorial, reflecting how the intersection of the micro, meso, and macro levels manifests in different households [1].

In this study, we examine how parents of preschool-aged children with obesity perceived changes in their eating, screen time and physical activity behaviours between the first and second waves of Covid-19 in three countries – Sweden, Romania, and Spain. This multinational design allows us to investigate whether different macro level contexts and policies have led to diverging manifestations of behaviour change in these households, and thereby highlight how the various components of children’s ecologies have interacted to produce obesogenic or protective behaviours during the pandemic. To our knowledge, this is the first study that attends to obesity-related behaviour changes between the first and second Covid-19 pandemic waves, while also accounting for changes in national policies and in household routines and capabilities.

## Methods

### Participant recruitment

The participants were parents recruited to a three-site randomised controlled trial (RCT) of an early childhood obesity intervention, conducted in Stockholm (Sweden), Timisoara (Romania), and Mallorca (Spain). The design of the RCT has been described previously [12]. In brief, the child had to be 2–6 years old, with overweight or obesity according to international criteria [13] no underlying medical condition(s) that may affect weight status, no previous experience of overweight or obesity treatment, and at least one parent able to communicate in Romanian, Spanish, or Swedish, respectively. The RCT was suspended from March 2020 due to the Covid-19 pandemic. Parents of children who were either on the waiting list or had already started the treatment were contacted by the researchers over the phone and asked if they were willing to participate in a semi-structured phone interview about the influence of the pandemic on their everyday lives. In Spain, 40 parents (in 40 families) were contacted; 10 declined due to unavailability, working long hours, or coping with an active Covid infection in their household. In Romania, 34 parents (in 34 families) were contacted; two declined due to lack of time or not wanting to participate, three were unavailable, and four dropped out of the study. In Sweden, 33 parents (in 31 families) were contacted; six parents were unavailable and two parents declined due to lack of time or not wanting to participate.

### Data collection

#### Wave 1

The interview guide was developed by the Swedish team under the supervision of KE. Interview questions were designed to allow the interviewed parents to describe how the pandemic affected them, the child, and the entire family. The questions captured pandemic-related disruptions in routines and treatment, for example: “How has the Coronavirus pandemic affected your family’s everyday life? Do you see any differences in your child? If so, can you describe these differences? Can you tell me about an activity you did with your child around food (cooking, eating together, etc.) that is different now compared to how it was before the pandemic?” For the full interview guide, please refer to supplementary file 1. Recognising that discussions of family life during the pandemic might be emotionally loaded, the questions were open-ended and worded in a neutral yet empathetic tone. This approach was highlighted as important during the pilot testing of the interview guide with three families in Sweden. After the pilot testing, the interview guide was translated to English and shared with the Spanish and Romanian teams; based on these teams’ comments, minor changes were made to the guide. The guide was then translated into Spanish and Romanian. Telephone interviews with a total of 80 caregivers in 78 families were conducted: 25 families (21 mothers, two grandmothers, one stepmother, and one foster mother) in Romania, 30 (28 mothers, one foster mother and one father) in Spain and 23 (13 mothers, eight fathers and both parents in two families) in Sweden. The interviews were conducted from mid-April through May 2020 in all sites.

#### Interim analysis

The Wave 1 interview data were analysed using inductive thematic analysis [14], with a phenomenological focus on parents’ perceptions and experiences [15]. Word processing software (Microsoft Word) was used throughout the analysis. First to analyse the data was the Romanian team (AC-E and IEJ-S, both paediatricians), which was also the first to have completed data collection. In their initial analysis, AC-E and IEJ-S identified open codes; these codes and interview quotes that supported them were then translated into English and discussed with study leaders PN, a professor of dietetics, nutrition, and food studies and KE, a medical anthropologist. Based on this discussion, the four authors developed a table with open code examples (e.g. ‘Child is walking more’), the higher order codes to which these open codes led (e.g., ‘More physical activity’), and the topic areas which described these higher order codes (e.g., ‘Physical activity’). To ensure comparative coding across the three sites, the authors shared this coding table with the Swedish and Spanish teams, who used this table to guide their coding processes. While the coding table provided guidance, it was not restrictive: in the Swedish and Spanish teams, researchers began their analysis process by familiarising themselves with the data and writing short descriptive summaries, independent of the coding tables. In the coding phase, the researchers were expected to add open codes unique to their interview sets and were free to add higher order codes as needed. Using this table, HRR and YVB then coded the Swedish and Spanish datasets, respectively, in consultation with PN and KE. When data from all three countries were analysed, PN and KE developed a summary of parental perceptions of change from before the pandemic to the first wave across four key domains: obesity-related changes, changes in the physical environment, changes in the emotional environment, and social and family-related changes. Of note, the initial codes were generated inductively, without being informed by ecological theory. It was only after these codes had been discussed among the team members that Bronfenbrenner’s ecological systems theory was applied to develop the analysis further.

#### Wave 2 – setting

When the interim analysis of the Wave 1 was complete, Europe was entering the second wave of the Covid-19 pandemic, heralding public health policy shifts and potential new challenges for the participating families. From September 2020, the RCT was conducted mostly online due to the pandemic. And by November 2020, about 6 months after our initial data collection, Romania, Spain, and Sweden had begun lockdowns of varying extents, ranging from a lockdown with school closure in Romania, to lockdowns where schools remained open in Spain and Sweden. In all three countries, children with any potentially Covid-related symptoms were subject to self-isolation restrictions, and indoor gatherings (such as in sports facilities) were limited. Whereas in Sweden, which remained open during Wave 1, the Wave 2 lockdown introduced new restrictions, in both Romania and Spain, the Wave 2 lockdowns were more flexible than the Wave 1 lockdowns, which featured strict restrictions on leaving the home, with police enforcement.

#### Wave 2 – data collection

Recognizing that the pandemic was a dynamic and long-term event, we decided our study should take a long view of obesity-related changes, and account for shifts between pandemic waves and how these might have affected the participating families. We therefore developed a follow-up study, with new interview questions about place of residence and work situation, reflecting the insights we gained from the interim analysis of the Wave 1 data. We also included questions similar to the ones we asked in the Wave 1 interviews, but these were designed to take the interviewees’ Wave 1 responses into account (e.g., “During the previous interview you mentioned that (name a few things about eating habits or food environment that the parent reported). Is this still the same or have you seen any change?”). We contacted all 78 families who participated in the Wave 1 interviews and asked if they would agree to take part in the follow-up interviews. Sixty-eight families (with a total of 70 caregivers) were interviewed again in November–December 2020: 23 families (19 mothers, two grandmothers, one stepmother, and one foster mother) in Romania, 25 (23 mothers, one foster mother and one father) in Spain and 20 (11 mothers, seven fathers and both parents in two families) in Sweden. All participants who took part in the Wave 2 interviews had also taken part in the Wave 1 interviews.

Data collected at baseline and follow-up were used to describe the population and to calculate child weight status at the time for the interviews. All participating families had filled out questionnaires regarding family background (child age and gender and parental age, gender, education level, employment status and weight and height). To describe the children’s weight status development between the interviews, available measurements on children’s weight and height within 4 months (pre or post) of the interviews were used. Children’s weight status (mean BMI z-scores) was derived using age and gender specific reference data [13]. Child BMI was used to categorize weight status as normal weight, overweight, obesity and severe obesity according to the international cut-offs [13].

### Ethics

The study was approved by the Ethics Committee of Scientific Research in University of Medicine and Pharmacy “Victor Babes”, Timisoara, Romania, October 31st, 2018 (25/31.10.2018), the Balearic Islands Ethics Committee, Mallorca, Spain, February 13th, 2019 (IB 3814/18 PI), and the Research Ethics Committee, Stockholm, Sweden, December 11th, 2018 (2018/2082–31/1). All parents/caregivers provided written informed consent for participation in the RCT, including consent to participate in a voluntary recorded interview. In both the Wave 1 and Wave 2 interview studies, all participating parents/caregivers, in addition to the written consent, were asked to provide an oral informed consent on record before the interview started.

### Analysis

The Wave 2 interview data were analysed in two stages. First, in each of the study sites, a local researcher (IEJ-S, CB, and UH for Romania, Spain, and Sweden, respectively) familiarised herself with the data, and then wrote an informal summary of the interview responses; this ensured that researchers were not entirely led by the template provided by the Wave 1 coding table. Each of the researchers then worked with the Wave 1 coding table, for a guided thematic analysis [14] that maintained the Wave 1 phenomenological emphasis on participants’ perceptions and experiences, as well as introduced a longitudinal qualitative approach (recurrent cross-sectional analysis) [16] to capture parents’ reflections on what had changed between Wave 1 and Wave 2. This enabled the comparison of codes across sites, whilst highlighting potential changes between Wave 1 and Wave 2; the researchers were free to refine the table to address issues specific to the current dataset. Then, the local researchers shared the English-language summaries and coding tables (with translated quotes or paraphrased interview segments) with KE and PN. Working comparatively across the three coded datasets, KE and PN developed a summary of findings parallel to the one developed for the Wave 1 data, highlighting the four key domains: obesity-related changes, changes in the physical environment, changes in the emotional environment, and social and family-related changes. The involvement of multiple authors in analysing the data locally and interpreting the findings across the three study sites ensured the robustness of the analysis in both the interim phase, following the Wave 1 interviews, and the final phase, following the Wave 2 interviews.

In the second stage of the analysis, KE reviewed the Wave two summaries and code tables (all of which utilized Microsoft Word) and tabulated the findings ecologically, case-by-case, using Excel. This process was guided by three key questions:

1. How have children’s obesity-related behaviours, as perceived by parents, changed between the first wave and the second?
2. How have parents’ practices changed (e.g., feeding, facilitating physical activity) between the first wave and the second?
3. From an ecological perspective, why have these changes occurred?

The ecological tabulation highlighted the micro (child, family, school, afterschool systems), meso (relationships between these systems), exo (changes parents experienced that did not directly relate to the children), and macro (policy and socioeconomic conditions) levels, with the following objectives:

- Identify what changed in the three key obesity-related behaviours (eating, physical activity and screen time) and in parents’ practices related to these behaviours;
- Identify what other changes occurred in these families’ lives (e.g. employment, social/emotional) in relation to macro-level processes (e.g. furlough, lockdown);
- Identify which changes were directly related by participating parents to their children’s obesity-related behaviours (e.g. school opened so the child is more active);
- Draw connections between other changes, which parents did not directly relate to their children’s eating, physical activity or screen time, and changes in obesity-related behaviours.

## Findings

### Sample description

The sample’s characteristics are described in Table 1, in total and by study site. In Sweden, the interviews were on average conducted 6.3 months apart (5.3 to 7.2 months). In Romania, 7.8 months (6.4 to 10.8 months) and in Spain 6.1 months (5.8 to 6.4 months).

**Table 1:** Characteristics of participating families

	*All*	*Sweden*	*Romania*	*Spain*
*Families*	*n = 78*	*n = 23*	*n = 25*	*n = 30*
Person interviewed, first interview, *n (%)*				
Mother	63 (81)	13 (56)	21 (84)	29 (97)
Father	9 (12)	8 (35)	–	1 (3)
Both parents	2 (3)	2 (9)	–	–
Other person	4 (5)	–	4 (16)	–
Person interviewed, second interview, *n (%)*	*n = 68*	*n = 20*	*n = 23*	*n = 25*
Mother	53 (12)	11 (55)	19 (83)	23 (92)
Father	8 (78)	7 (35)	–	1 (4)
Both parents	2 (3)	2 (10)	–	–
Other person	5 (7)	–	4 (17)	1 (4)
*Child*				
Gender (girl), *n (%)*	47 (60)	15 (65)	13 (52)	19 (63)
Age at interview 1 (years), *mean (SD)*	5.5 (1.3)	4.8 (1.2)	5.8 (1.0)	5.7 (1.5)
**Interview 1**				
Child weight and height measures, derived within ±4 months of the interview at, *n (%)*	*n = 67*	*n = 22*	*n = 25*	*n = 20*
Baseline or close to treatment start	9 (14)	9 (41)	–	–
After three to six months of treatment	23 (34)	6 (27)	10 (40)	7 (35)
After nine to 11 months of treatment	27 (40)	7 (32)	11 (44)	9 (45)
After 15 months of treatment	4 (6)	–	–	4 (20)
Measured outside the study - had not received treatment	4 (6)	–	4 (16)	–
Weight status, *n (%)*				
Normal weight	1 (2)	–	1 (4)	–
Overweight	10 (15)	6 (27)	2 (8)	2 (10)
Obesity	25 (37)	9 (41)	9 (36)	7 (35)
Severe obesity	31 (46)	7 (32)	13 (52)	11 (55)
BMI z-score*, mean (SD)*	3.1 (1.1)	2.7 (0.8)	3.1 (1.3)	3.4 (1.0)
**Interview 2**				
Child weight and height measures, derived within ±4 months of the interview, *n (%)*	*n = 59*	*n = 20*	*n = 23*	*n = 16*
After three to six months of treatment	8 (13)	6 (30)	2 (9)	–
After nine to 11 months of treatment	21 (35)	8 (40)	6 (26)	7 (44)
After 15 to 17 months of treatment	23 (38)	6 (30)	11 (48)	6 (38)
After 21 months of treatment	4 (7)	–	–	3 (19)
Measured outside the study - had not received treatment	4 (7)	–	4 (17)	–
Weight status, *n (%)*				
Normal weight	3 (5)	1 (5)	2 (9)	–
Overweight	8 (14)	6 (30)	1 (4)	1 (6)
Obesity	19 (32)	9 (45)	9 (39)	1 (6)
Severe obesity	29 (49)	4 (20)	11 (48)	14 (88)
BMI z-score*, mean (SD)*	3.0 (1.0)	2.5 (0.9)	2.9 (1.1)	3.5 (0.8)
Change in BMI z-score between interview 1 and 2, *mean (SD)*	- 0.06 (0.7)	- 0.1 (0.6)	- 0.2 (0.7)	0.18 (0.5)
*Mother*				
Age (years), *mean (SD)*	*n = 71*	*n = 21*	*n = 21*	*n = 29*
	38.4 (5.7)	38.7 (6.3)	37.1 (4.8)	39.3 (5.8)
Born abroad, *n (%)*	*n = 72*	*n = 21*	*n = 24*	*n = 27*
	23 (32)	15 (71)	0	8 (30)
Weight status, *n (%)*	*n = 68*	*n = 19*	*n = 22*	*n = 27*
Normal weight	18 (26)	7 (37)	4 (18)	7 (26)
Overweight	29 (43)	8 (42)	13 (59)	8 (30)
Obesity	14 (21)	3 (16)	3 (14)	8 (30)
Severe obesity	7 (10)	1 (5)	5 (9)	4 (14)
Education level, *n (%)*	*n = 72*	*n = 21*	*n = 22*	*n = 29*
University degree	38 (53)	11 (52)	15 (68)	12 (41)
Senior high school diploma	14 (19)	5 (24)	4 (18)	5 (17)
Vocational diploma	11 (15)	3 (14)	2 (9)	6 (21)
Finished compulsory school	9 (13)	2 (10)	1 (5)	6 (21)
Employment, *n (%)*	*n = 70*	*n = 21*	*n = 22*	*n = 27*
Full-time	25 (36)	13 (61)	9 (41)	3 (11)
Part-time	26 (37)	3 (14)	5 (23)	18 (67)
Student	2 (3)	2 (10)	–	–
Parental leave/sick leave	4 (6)	1 (5)	3 (14)	–
Unemployed	5 (7)	2 (10)	1 (5)	2 (7)
Other	8 (11)	–	4 (17)	4 (15)
*Father*				
Age (years), m*ean (SD)*	*n = 68*	*n = 23*	*n = 17*	*n = 28*
	41.0 (5.7)	40.7 (6.7)	39.6 (6.1)	42.1 (4.5)
Born abroad, *n (%)*	*n = 64*	*n = 22*	*n = 17*	*n = 25*
	18 (28)	13 (59)	0 (0)	5 (20)
Weight status, *n (%)*	*n = 64*	*(n = 22)*	*(n = 16)*	*(n = 26)*
Normal weight	9 (14)	7 (32)	2 (12.5)	–
Overweight	25 (39)	7 (32)	4 (25)	14 (54)
Obesity	19 (30)	4 (18)	8 (50)	7 (27)
Severe obesity	11 (17)	4 (18)	2 (12.5)	5 (19)
Education level, *n (%)*	*n = 67*	*(n = 22)*	*(n = 17)*	*(n = 28)*
University degree	26 (39)	12 (55)	6 (35)	8 (29)
Senior high school diploma	13 (19)	4 (18)	4 (24)	5 (18)
Vocational diploma	10 (15)	4 (18)	2 (12)	4 (14)
Finished compulsory school	14 (21)	2 (9)	5 (29)	7 (25)
Primary school	4 (6)	–	–	4 (14)
Employment, *n (%)*	*(n = 66)*	*(n = 21)*	*(n = 17)*	*(n = 28)*
Full-time	46 (70)	19 (90)	15 (88)	12 (43)
Part-time	12 (18)	1 (5)	1 (6)	10 (36)
Parental leave/Sick leave	2 (3)	1 (5)	1 (6)	–
Unemployed	2 (3)	–	–	2 (7)
Other	4 (6)	–	–	4 (14)

Persons interviewed other than parents were grandmother, stepmother and foster parents. In two Swedish families both parents were interviewed. Child weight status was calculated based on available weight and height measurements performed at the most 4 months before or after the interviews. Children’s weight status was classified as normal weight, overweight, obesity and severe obesity according to international age and gender adjusted cut-offs for BMI (International Obesity Task Force, IOTF). The same weight status classifications for BMI z-scores are, for boys > −1.01 < 1.31; ≥ 1.31 < 2.29; ≥ 2.29 ≤ 2.93 and ≥ 2.93 respectively, and for girls > −0.98 < 1.24; ≥ 1.24 < 2.19; ≥ 2.19 < 2.82 and ≥ 2.82 respectively. Parental characteristics are available data from baseline. The parents’ weight status was classified as normal weight (> 18.5 < 25), overweight (≥ 25 < 30), obesity (≥ 30 < 35) and severe obesity (≥ 35) according to the World Health Organization’s reference values for BMI. Other forms of occupation statuses mentioned were seasonal work and housewife

Weight status measurements of 10 children from the Spanish sample were excluded as they were taken more than 4 months before or after interview 1. For interview 2 one additional child’s measurement was excluded for the same reason. Other missing values were due to incomplete data collection

*Abbreviations*: *SD* standard deviation, *BMI* Body mass index

Among the 78 interviewed families (80 legal guardians), 68 families were interviewed twice. More fathers were interviewed in the Swedish sample and more of the Swedish families had a foreign background (i.e., the parent having been born in another country) as compared to both Romania and Spain. In the total sample, the mean age of mothers was 38.4 years, 32% were born abroad, 53% had a university degree, and 27, 43, and 31% were classified as having a normal weight, overweight, and obesity or severe obesity, respectively. For fathers, the mean age was 41.0 years, 28% were born abroad, 39% had a university degree, and 14, 39 and 47% were classified as having a normal weight, overweight, and obesity or severe obesity, respectively. The children (*n* = 78) were on average 5.5 years old at the time for the first interview. Among the children with parents who were interviewed twice, 59 children had valid measures at the time of the first Interview; 2% (*n* = 1) were classified as having a normal weight, 15% (*n* = 9) overweight, 39% (*n* = 23) obesity and 44% (*n* = 26) severe obesity. At the time for the second interview, 58 children had valid measures; 5% (n = 2) were classified as having a normal weight, 14% (*n* = 8) overweight, 33% (*n* = 19) obesity and 50% (*n* = 29) severe obesity. Child weight status body mass index (BMI) z-score was 3.1 at the time for the first interview and 3.0 at the time for the second interview and mean change in weight status was − 0.06 between the two interviews. How long families had participated in the study differed at the time of the interviews; thus, the timepoints when child weight status was measured differed as well (see Table 1). However, the available data show that, in Sweden and Romania, participating families seem to have managed to continue the child’s weight management, as shown by the children’s improved weight status.

In the manuscript, participant anonymized codes are provided parenthetically when direct quotes are shared. The codes are start with a letter (M = mother, F = father, GM = grandmother) followed a four-digit number, where the first digit represents country of interview (1 = Sweden, 2 = Romania, and 3 = Spain) and the last three represent the participant’s ID.

## Themes

Four themes were developed through qualitative longitudinal analysis: (1) Children’s diverging directions of behaviour change; (2) Parents’ responses to the pandemic; (3) Emotional and social resources; (4) Household resilience.

### Theme 1: Children’s diverging directions of behaviour change

Across the three study sites, parents’ reports about children’s obesity-related behaviours diverged markedly. While some children experienced positive changes between the first and the second pandemic waves, others experienced negative changes. For example, when asked about their children’s physical activity, the following three mothers offered substantially different responses – whereas a mother from Spain said her child was more physically active than during lockdown but less active than before the pandemic, a mother from Romania said her daughter grew used to sedentary behaviours, and a mother from Sweden said her child’s interest in physical activity increased, though this was related to her growing up, rather than to the pandemic:

The activity has increased because they do physical education at school, but before the pandemic, [the child] used to go dancing twice a week and we have not done this again. (M3001)

My child no longer has that vitality of children… usually they run, scream; she is much quieter, more lazy, she got used to sitting at school, doing homework and so on, not doing much effort. (M2014)

Somehow, she seems to play more by herself and is playing with her sister… she has a little sister who is almost two years old, so then it became interesting to play with her. Another thing, I think she has lost weight, so now she has more energy to run and jump and has more energy, not just sit and watch [the screen]. (M1068)

Similarly divergent accounts arose in response to questions about children’s food choices and eating behaviours. Many parents, like this mother from Spain, reported their children engaged in emotional eating:

She is more anxious and wants to eat at all hours, but I have been able to introduce more fruit and new foods, for example, turkey. (M3051)

Some parents, however, reported no change in children’s lifestyles and habits between the first and the second waves, while others said their children’s eating improved. For example, a mother from Sweden (M1005) said her son had learned to recognize satiety and was less preoccupied with food.

Screen time behaviours likewise differed between families. The great majority of Spanish parents reported their children engaged in less screen time compared to the first wave, due to the opening of schools and the closure of online education. However, some parents in the Swedish and Romanian samples said their children increased their use of screens, albeit for different reasons. A father from Sweden (F1071) said his children engaged in less screen time, because of other activities at home like reading and playing, implying that screen time reduction was directly related to the increase in home-based leisure activities. In contrast, a mother from Romania said her child engaged in more screen time due to online school, but that he chose to spend his playtime engaged in other activities:

The time spent in front of the screens is considerably more. But what I can say is that, since he attends online school, he seems to have developed a bit of a repulsion [towards the tablet] (…). He doesn’t spend that much time with the tablet anymore. (M2003)

### Theme 2: Parents’ responses to the pandemic

Changes in children’s obesity-related behaviours, particularly eating and physical activity, were linked to changes in how parents responded to the challenges presented by the pandemic and their children’s reactions to these challenges. Across the three study sites, parents reported diverging responses. As mentioned in Theme 1, many of the parents said their children craved comfort foods and asked for treats. However, their responses differed. For example, one mother from Romania, who said her daughter gained weight due to stress, eating and snacking, responded by ending home baking:

We try to avoid white bread, and homemade bread, because last spring, we started making bread at home and it smelled all over the house and she was eating almost the entire bread core on her own. (M2014)

In contrast, a mother from Sweden (M1026), who said her daughter wanted treats and acted out if she did not receive food, responded by increasing home cooking. She said that working from home enabled her to “cook between some meetings, between some jobs you have to do, you start a bolognaise, you start something in the oven, so that it’s ready for the evening. So, it’s much more homemade food [served in the home]”. It also allowed her to collect her children from school earlier, meaning that they did “not have to wait around and be hungry”.

Other parents said they ‘gave in’ to their children’s food demands, rather than negotiating or regulating them. This was often linked to expressions of overwhelming stress. A mother from Sweden, whose son was self-isolating with cold symptoms, said her son was constantly asking her for food, and explained she did not have the energy to resist:

It is very hard to handle. He has constant cravings for something [to eat]. And all the time we have to check what he wants. And I really try to avoid sweets as much as possible, but, you know, the days are long, morning to night and all the time… There's been some candy here and there and salty snacks here and some popcorn there, just to keep him sitting still for a bit. (M1063)

Whereas parents’ main challenge in regulating children’s eating behaviours was in responding to their increased appetites and desire for comfort foods, their main challenge in facilitating physical activity was finding creative solutions when faced with the closure of schools and indoor sports facilities. For parents in the Swedish subsample, this was a particular challenge as the closure of sports facilities was introduced in the second wave. This proved to be a barrier to some activities, as one mother (M1051) explained, her child took up swimming instead of football, but could not progress to an intermediate swimming class as all indoor public pools had closed. Some parents mitigated these barriers by facilitating alternative physical activity outdoors. For example, one father (F1066) said, “we try to be outdoors with them as much as before. It is [now] usually calmer in the playgrounds…”, while a mother said she and her partner took turns going outside with the children to facilitate their activity:

…we still walk to the school and back, or ride a bike or a kick-bike or something like that and I think that because both I and (partner's name) work from home, we also have a great need to get out on the weekends. So maybe we are out a little more due to Corona because you feel like you have a need to be outside more. … And when one parent sits and does homework with (one child), the other (parent) tries as a routine to do some physical activity with (the other child), go out and kick a ball, or throw a frisbee or something like that. (M1005)

Additionally, for this mother, as for other parents who lived in houses, access to backyards was an important element of facilitating physical activity at home. This, however, was impossible for parents who lived in flats, even where there was access to shared courtyards. One mother described the difficulties she faced when trying to facilitate her son’s physical activity inside their flat as well as in the courtyard:

We have a neighbour who is sick or so, I think it's mentally, because when (child's name) moves around a little, or runs, [then] she [the neighbour] knocks on the ceiling all the time, so we try to walk slowly or just sit, do something that doesn’t involve moving around as much…. even when we are in the yard she shouts, shouts at them “Can you be quiet!” (M1030)

Having no access to yards made encouraging children’s physical activity particularly problematic. A father (F1064) said he felt guilty for not facilitating children’s activities at home, particularly since his daughter had to quit dancing and he could not take the children swimming due to his vulnerability to Covid.

In Romania and Spain, where restrictions on physical activity had eased from the first to the second wave, parents described differently how they facilitated physical activity. In the Spanish subsample, most parents focused on the availability of gardens and yards as enabling physical activity during the first wave’s lockdown, with many saying that after lockdown the yard diminished in importance as their children became more active at school and elsewhere. However, few parents spoke directly about facilitating their children’s physical activity (e.g., by exercising together) beyond providing them access to an outdoors space, whether domestic (yard) or public (playground). This might have reflected weather-related differences between the sites: whereas in Sweden and Romania parents spoke about the challenges of facilitating physical activity in cold winter weather, this was not a concern in the Spanish sample. In the Romanian subsample, parents emphasized the renewed importance of yards, with the second wave’s closure of schools and sports facilities; accordingly, parents who lived in apartment buildings said they felt disadvantaged. Parents also described trying to find replacements for indoor sports and school-based physical education. This, however, proved challenging in cold weather, as one grandmother described:

In August, September and at the beginning of October, I took him swimming outdoors. But as time went on, it became too cold, they [the coaches] could no longer make the children swim in the pool outside. They did not have access to a space, so they were exercising outside, as much as possible. Finally, they received a room [for indoor exercises]. He went there for a while, but the coaches and probably also the parents were not satisfied, only doing these strength exercises, push-ups, knee bends, running... so they decided to move again. I couldn't take him there, we don't have a car. Before, one of the coaches who lived in the area, was coming to take him to swim. I went with him for a few weeks, but then I couldn't, it was too difficult for me. (GM2012)

### Theme 3: Emotional and social resources

Changes in parents’ facilitation of healthy eating and physical activity were nested in the household’s emotional and social environment. Across the study sites, parents who described pandemic measures in positive terms – for example, as allowing more time for family, and reducing job and commuting stress – also tended to describe handling their children’s eating and physical activity with greater ease. Similarly, parents who described the pandemic and/or pandemic measures in negative terms – for example, as leaving them feeling isolated, overwhelmed with stress, or anxious about infection – also tended to describe difficulties in handling their children’s eating and physical activity.

A frequent source of both joy and conflict was the intense togetherness of the household ‘bubble’. Many parents described spending more time together with their children due to working from home, being placed on furlough, or becoming unemployed, combined with school closures and/or self-isolation measures. Some parents said this newfound family togetherness allowed them to develop stronger emotional bonds with their children, while others described it as stressful. One mother from Romania explained that the advantages and disadvantages of forced togetherness could coexist. The lockdown, she said, enhanced the family’s sense of closeness, as both parents worked from home:

I think we are closer to each other, in the sense that we do family meetings, as my child likes to say, she comes to us very often, she just wants to talk to us, not necessarily that something happened, or that there is a problem in her life, but simply socializing and debating certain things. (M2014)

At the same time, this mother also found the unrelenting socializing with her daughter difficult:

It is not easy to stay non-stop with the child at home, neither for the child, nor for the adults; at a certain moment my little girl said to me ‘mother I am tired of you, I love you madly, but I am tired of you, I want to do something else’.” (M2014)

For this mother, as for others, the stress of intense togetherness was connected to a sense of social isolation.

Our family life has been greatly affected in several aspects: emotional, eating behaviour and many others. We got isolated. We didn’t interact with others physically; we have just stayed in the family. We struggle with the online school, we aren’t going anywhere. (M2014)

Many parents reported they missed socializing with their family and friends; they also reported their children missed their classmates and grandparents. For some parents, this meant making the decision to socialize, despite pandemic restrictions, while other parents waited for the easing of restrictions. Across the sample, parents considered renewed socializing, and particularly the re-entry of grandparents into their families’ lives, an important means of improving their own and their children’s wellbeing.

Another aspect of the household ‘bubble’ was the increased pressure on parents, and particularly mothers, to perform as employees, educators, and caregivers. This was particularly an issue in Romania, where schools had closed again with the second wave, as described by one mother:

It was hard for me to distribute time; to make cards for kindergarten [as a teacher]… to be a farmer and a cook [for my family]... (M2035)

However, even in Sweden, where schools remained open, and in Spain, where schools reopened after the first wave’s lockdown, parents described being stressed over the prospect of their child having to self-isolate due to Covid symptoms or after being in contact with an infected friend. Because many parents continued working from home, this meant that their workday could be interrupted at a moment’s notice, as captured in the following quotes by a mother from Spain and a father from Sweden, respectively:

Interviewer: What is the biggest change your family has experienced since we last spoke?

Participant: The fact of not being able to commit to a job or to people in case the girls have to self-isolate due to some contagion at school. (M3007)

… so, we [have] two children going to preschool, they are sent home, then I have to stay home, then in March and May, I think I was more at home than at work. (F1064)

Family support was key to managing these uncertainties. As a mother from Sweden described, sharing parenting responsibilities allowed her to navigate the children’s self-isolation periods:

In preschool they are stricter than normal [requiring self-isolation] with a few symptoms [of Covid]. (…) it gets a little harder, but because we share with my husband, he is also at home, then it works. It is possible that you are a little stressed for half the day, but the other half it is a little calmer when he is with [the children]. (M1068)

Beyond the day-to-day stresses of parenting in a pandemic without social support, parents expressed stress and uncertainty about the future. A mother from Sweden (M1026) said her daughter worried she may never see her grandparents again. Others, including a mother (M1030) and a father (F1071) from Sweden and a mother from Spain (M3011), worried they or their clinically vulnerable children might become infected with Covid. And still others were worried life may never be the same again. As one father from Sweden, weary of facing the unknown, asked:

For how long will this continue? (F1005)

### Theme 4: Household resilience

Parents’ approaches to managing their children’s eating and physical activity, as well as their experiences of emotional and social wellbeing or distress, were closely linked with pandemic-related regulations. Restrictions on socializing, school, and sports facilities closures and reopenings, self-isolation measures, home working and furlough all had an impact on how households negotiated the pandemic. However, the resources and responses parents described when facing these macro-level changes were ultimately linked to household resilience. Across the three study sites, parents whose households faced insecurity, particularly in the form of potential or actual job loss, were most affected by the changes in pandemic-related regulations.

Going back to the parents whose quotes were introduced in the first three themes, their reported experiences can be contextualized within insecurity and resilience. A mother from Spain, who reported that her child was craving more food, also said she managed these cravings by providing him fruit and new foods. With her husband now working from home, and with her own work as a stay-at-home mother, she felt that they were able to spend more time together and be more relaxed:

It influences in a positive way. Because this way we all spend more time together, the family environment is much better, we go out together and do everything as a family. On the subject of food, I am still in control. (M3051)

In contrast, a mother from Sweden, who was on sick leave and whose husband worked 13-hour days outside the home, said she felt too overwhelmed to negotiate her son’s food cravings:

… now we are home for long days, he is whiny, he wants to go to kindergarten, he cannot. So, it's really hard. So, he asks for something to eat, right… I try to avoid as much as possible, but some days, it gets too annoying, and I do [give in]. No. And I don’t have the energy. (M1063)

Similarly, a mother from Sweden (M1005) who worked at home with her husband and found this arrangement less stressful than before the pandemic, found it easy to facilitate her child’s physical activity, whereas a father from Sweden (F1064), who was clinically vulnerable and anxious about potentially losing his job, felt it was impossible for him to pursue physical activity opportunities for his children.

## Discussion

This study is the first to explore parents’ perceptions of changes in their children’s eating, physical activity, and screen time behaviours between the first two waves of the Covid-19 pandemic in Europe. Using an ecological approach, we analysed interviews with parents of preschool-age children with obesity in Spain, Romania, and Sweden. Our analysis found that, across the three study sites, parents’ reports about changes in children’s obesity-related behaviours diverged markedly. Although all children began the study with overweight or obesity, they experienced the pandemic differently, with some engaging in healthier eating and physical activity, and others engaging in comfort eating and a more sedentary lifestyle. These differences in children’s behaviours were closely related to differences in parents’ feeding and activity facilitation practices. Some parents reported feeling overwhelmed and unable to negotiate the challenges posed by the pandemic and their children’s responses to it, whereas others described successful strategies that helped their children practice healthy eating and become more physically active. Importantly, parents who reported greater emotional and social distress, as a result of the pandemic, also said they found it difficult to manage their children’s eating and activity, while those who described pandemic measures as positively impacting on their stress levels (e.g., by allowing them to spend more time with their family) also described healthy feeding and activity as easier. The intersection of feeding and physical activity facilitation with emotional and social resources was further embedded in overall household resilience. Parents who had secure employment and financial/housing resources were better able to adapt to the challenges posed by the pandemic. In contrast, parents who experienced job insecurity – in many cases, due to the pandemic – found it difficult to cope with eating- and activity-related changes. In a number of cases, this socioeconomic vulnerability was compounded by clinical vulnerability to Covid-19, leading to feelings of anxiety and chronic stress. This finding was consistent across the three study sites, suggesting that household resilience is central to children’s healthy eating and physical activity, regardless of differences in policy responses to the pandemic. Thus, although policy – such as the closure of schools and sports facilities – made a difference, how families adapted to it depended on the emotional and material resources they had.

Our finding that parents’ emotional and social resources were linked to their capacity to facilitate children’s healthy eating and physical activity is consistent with the family ecology literature on childhood obesity. This literature, which has investigated how family contexts interact with community, organisational, and other contexts in childhood obesity, has found that parents who experience chronic stress and lack of social support have reduced ability to support their children in adopting healthy eating, physical activity and screen time behaviours [17]. Along similar lines, studies focusing on the ecologies of lower-income families found that those parents who experience fewer stressors and have greater social support are also able to reduce children’s screen time and increase their physical activity [18, 19]. Notably, however, the extant literature on family ecologies has yet to explore rapidly changing social contexts – such as those families encountered during the Covid-19 pandemic – which may impact on parents’ emotional and social wellbeing in sudden and unpredictable ways.

A key contribution of our study is the finding that household resilience was closely related to how families adapted to the Covid-19 pandemic and its food- and activity-related consequences. The childhood obesity literature has emphasized family resilience – e.g., family structure, coherence, and routines – as central in mitigating the impact of adverse childhood experiences and environmental stressors on children’s weight status [20–22]. Our study, however, suggests that family resilience is embedded within the overall resilience of the household. This finding is consistent with large-scale quantitative research, which found that obesity is closely associated with economic insecurity, suggesting that obesity may be a chronic stress response [23, 24]. It is also consistent with emerging psychological research conducted during the Covid-19 pandemic, which found that, in the presence of socioeconomic insecurity, parenting stress was associated with children’s emotional dysregulation, children’s increased screen time, and parents’ non-responsive feeding [25–27]. Of note, while mentions of household resilience in the childhood obesity literature have exclusively been in relation to food security (e.g. [28], ,our study suggests that household resilience impacts on childhood obesity via the management of everyday insecurity not directly related to food, such as employment and clinical vulnerability.

The findings suggest that, as childhood obesity treatment programs adapt to the Covid-19 pandemic, the advice provided to parents should take into account differences between the capabilities of households in responding to the challenges posed by the pandemic and policy measures that address it. Although SES is a key factor in childhood obesity, how SES influences families’ willingness and ability to engage in treatment is rarely measured [29, 30]. Treatment providers should ask parents about challenges their households have experienced during the pandemic, including those not directly related to children’s eating and physical activity, such as changes in economic and housing security, as well as clinical vulnerability. Understanding household resilience and vulnerability will allow treatment providers to explore what is possible and tailor advice to individual households, and thereby empower parents to enact positive change even in difficult circumstances.

The study has several notable strengths. The sample was drawn from an international RCT for the families of young children with weight excess, allowing us to focus on families whose children had similar weight status. At the same time, by recruiting participants from three countries with different Covid-19 pandemic responses, the study could address how policy might account for potential differences in the experiences of families with children with obesity. Moreover, the high participant response rate in both the Wave 1 and Wave 2 interviews and the low dropout rate, with 68 families participating in both interviews, meant that the sample was diverse, including parents from different socioeconomic groups, thereby enabling an analysis of participants’ responses in light of differences between households. In addition, the study’s follow-up design allowed us to adapt the interview questions to each individual participant, thereby increasing the relevance and continuity of data collection between the Wave 1 and Wave 2 interviews. The study had some limitations. By necessity, the study was limited by its focus on parents’ perceptions, and we cannot be sure that what parents reported reflected accurately what happened in practice. An additional limitation was the study’s focus on parents who had enrolled in a childhood obesity RCT, as their perceptions may differ from those of parents whose children have obesity but who have not enrolled in treatment. Finally, as in the majority of studies on childhood obesity, mothers greatly outnumbered fathers. Recent research on family treatment for childhood obesity has suggested that fathers’ and mothers’ experiences of and responses to obesity treatment may differ in important ways [31]. Although the number of fathers in our sample was too small to investigate gender-related differences in parents’ perceptions of children’s obesity-related behaviours during the pandemic, future research should attempt to include more fathers in order to account for these potential differences.

## Conclusion

Using an ecological approach, this study was the first to explore how parents perceived changes in their children’s eating, physical activity and screen time behaviours between the first two waves of the Covid-19 pandemic in Romania, Spain and Sweden. The study found that, although all participants were recruited from an RCT for families of children with obesity, they reported markedly different responses to the second wave of the pandemic, with some children engaging in healthier eating and physical activity, and others engaging in comfort eating and a more sedentary lifestyle. Notably, differences in children’s obesity-related behaviours were closely related to differences in parents’ feeding and physical activity facilitation practices, which were, in turn, linked to parents’ emotional and social wellbeing. Moreover, across all study sites, despite differences in national policy, parents’ feeding, and physical activity facilitation practices as well as their emotional and social wellbeing were embedded in household resilience. In resilient households, where parents had secure housing and employment, they were better able to adapt to the challenges posed by the pandemic and the policy responses to it, whereas parents who experienced household insecurity found it more difficult to cope. As the Covid-19 pandemic is turning into a long-term public health challenge, studies such as ours are crucial for developing effective prevention and treatment responses in the childhood obesity field.

## Supplementary Information

**Additional file 1.**

## Data Availability

All data generated or analysed during this study are included in this published article [and its supplementary information files].
