# Supplementary material for: Explaining the complex impact of the Covid-19 pandemic on children with overweight and obesity: a comparative ecological analysis of parents’ perceptions in three countries
Source: BMC Public Health. 2022 May 17;22:1000. doi: 10.1186/s12889-022-13351-1 (PMC9113066; doi:10.1186/s12889-022-13351-1)
Supplement: Supplementary file 1 — Additional file 1. [file 12889_2022_13351_MOESM1_ESM.docx]

**Interview guide W1**

**Introduction**

Hi, I’m calling from the More and Less study that you are participating in. Do you have some time to talk? As the Coronavirus has affected the everyday life for many families, we would like to interview some of you for how it has affected your family. The interview would take about 20-25 minutes and would be conducted over the phone. This will be part of our study of what facilitates and what hinders healthy weight development. Would you be willing to participate? If now is not a good time, we can find another time that suits you. If it isn’t possible for you to participate, would it be ok if I ask the other parent?

*If yes:* As I said earlier (when we spoke earlier), because of the unique situation we are in because of the Coronavirus, I would like to ask you some questions about how the situation has affected your family. Since the More and Less study is about how we can help children to a healthy weight development, I am also curious about your thoughts on how the Corona virus has affected you from that perspective. I will ask questions on how the situation is now, how it was before and I will also ask you to think ahead. You can decide how much and what you want to tell me. If there are any questions you prefer not to answer, that’s perfectly fine. Does this sound ok?

In order for me to remember everything we talked about; I will record the conversation so that I can later write down what we have discussed. No one outside of our research group will know who you and your family are. Do you have any questions on the information you have received about this interview before we start?

Since this is a part of the study, I would like to ask you to answer the question: Do you agree to participate in this interview? And are you happy for me to record the interview?

**Then I will begin with the questions. There are many things that affect families right now.**

1. So, broadly speaking, how has the Coronavirus pandemic affected your family’s everyday life?
   1. In regard to: preschool/work, routines at home, stress levels (your own and your child’s)?
   2. What is the biggest change your family has experienced?
2. What would you say has been most difficult to handle?
   1. Is there anything in particular that you are concerned about?
3. Do you see any differences in your child? If so, can you describe these differences?

**And now to the small tasks and habits in everyday life …**

1. Thinking about this past week, can you tell me about an activity you did with your child around food shopping, cooking, or eating that is different now compared to how it was before the Corona pandemic?
   1. Follow up: can you tell me a bit more about it?
   2. Follow up: What was it like before?
   3. Follow up: Is there something else that is different now?
2. Thinking about this past week, can you tell me about an activity you did with your child around physical activity that is different now compared to how it was before the Corona pandemic? (This may include exercise, play, and other ways of staying active at home or outdoors but also screen time.)
   1. Follow up: can you tell me a bit more about it?
   2. Follow up: What was it like before?
   3. Follow up: Is there something else that is different now?
3. So summing up, you have described things you do differently now, do you feel that these activities have become new habits in your family?
   1. How do you feel about these new habits?
      1. (If answered positively or ambivalently) Would you like to/would it be possible for you to keep any of these new habits after the pandemic?
         1. (If further probing needed) Would you like to keep any of your family’s new food and meal habits?
         2. (If further probing needed) Would you like to keep any of your family’s new physical activity and screen time habits?
         3. Are there any habits you’d prefer not to keep?
      2. (If answered negatively) Are you worried that these new habits might stay in place after the Coronavirus situation?

**Ok, let’s move on. Now I thought we could talk about the reason why you wanted to participate in the More and Less study?**

1. When you signed up for the study, what did you want help with?
   1. Has this changed since the Corona crisis?
2. Who in health care have you been in contact regarding the child’s weight before the Corona outbreak?
   1. Can you estimate how frequently you’ve been in contact? (This can include in-person visits, phone calls, etc.).
   2. What help did you receive that has helped you now?
   3. If no health care contact, what would have been helpful in the situation we are in now?
3. a) Thinking back on the support you have received since starting the study, parent groups/standard treatment. Is there anything that you learnt or talked about during the treatment/parent sessions that you can use and has been helpful during the pandemic?
   - 1. Follow up: Can you give examples?
     2. Follow up: Are there any parenting tools that you have tried that helped you in this situation?
     3. Thinking about the current situation is there something that you think we should add to the program?

8 b) Regarding the MINISTOP app. Would you say that you have used it more or less
 frequently during the pandemic/quarantine?

I. Follow up: Were there any functions in the app that you appreciated more with time?
II. Follow up: Did the advice in the app help you during the pandemic situation. Can you give an example?

1. Have you had any contact with this health care professional (or another health care professional) regarding the child’s weight since the beginning of the Coronavirus pandemic? Why or why not?
   1. If yes, what kind of help have you received? Have you received advice that helped you with the current situation? If so, could you tell us a bit about this advice?
   2. (If they indicate they didn’t have contact because of reduced healthcare capacity or other factors related to the pandemic) What kind of help did you need and couldn’t receive?
2. Is there anything you would like to add or bring up that we haven’t discussed yet?

**Examples of follow-up questions during the interview:**

- Could you develop that…
- Can you explain that more in detail…
- What do you mean when you say…
- That sounds interesting, tell me more about that!
- You mentioned something about… can you tell me more about that?
- In what way?
- How do you handle this?
- Can you give me an example?
- Have I understood you correctly if you mean that…
- I would like to go back to what you said about…
- What did it mean to you?
- Can you describe more about that…

**Interview guide W2 - follow up**

**Introduction**

Hi, my name is … and I’m calling from the More and Less study. Do you have some time to talk? In April (or May) you kindly participated in an interview about how the Coronavirus affected your family. We would like to ask you how you have been since then. The interview would take about 20-25 minutes and would be conducted over the phone. This will be part of our study of what facilitates and what hinders healthy weight development. Would you be willing to participate? If now is not a good time, we can find another time that suits you. If it isn’t possible for you to participate, would it be ok if I ask the other parent?

*If yes:* We spoke in the Spring because of the unique situation in which we were then, due to the Coronavirus. As the Coronavirus pandemic continues to affect our lives now, I would like to ask you a few questions about how the situation has been affecting your family in the last few months. These questions are similar to the ones you responded to in the Spring. Since the More and Less study is about how we can help children to a healthy weight development, I am also curious about your thoughts on how the Corona virus has affected you from that perspective. I will ask questions on how the situation is now, how it was during the last 6 months and I will also ask you to think ahead. You can decide how much and what you want to tell me. If there are any questions you prefer not to answer, that’s perfectly fine. Does this sound ok?

In order for me to remember everything we talked about; I will record the conversation so that I can later write down what we have discussed. No one outside of our research group will know who you and your family are. Do you have any questions on the information you have received about this interview before we start?

Since this is a part of the study *(start recording)*, I would like to ask you to answer the question: Do you agree to participate in this interview? *(Wait for participant to answer this question before asking the second question)*. And are you happy for me to record the interview?

**Then I will begin with the questions; when you answer these, please think about your family’s experiences in the last six months.**

1. So, broadly speaking, how has the Coronavirus pandemic affected your family’s everyday life since we spoke in the Spring?
   1. In regard to: preschool/work, routines at home, stress levels (your own and your child’s)?
   2. What is the biggest change your family has experienced since we spoke last?
2. What would you say has been most difficult to handle?
   1. Is there anything in particular that you are or have been concerned about?
3. Have you noticed any differences in your child over the last 6 months? If so, can you describe these differences?
   1. Prompts: have you noticed any differences in your child’s activities? Eating behaviours? Other behaviours?
4. During the previous interview you mentioned that (remind about few things about **eating habits or food environment** that the parent reported) Is this still the same or have you seen any change?
5. During the previous interview you mentioned that (remind about few things about **physical activity/screen time** that the parent reported) Is this still the same or have you seen any change?
6. Are you living in the same place where you resided when we spoke last?
   1. Is this a house or a flat?
   2. Do you have a garden?
   3. Do you live in the countryside or in the city?
   4. We are asking these questions to understand whether different home environments have affected children’s physical activity patterns during the Coronavirus situation. Do you think your home environment has had an impact on your child’s physical activity in the last six months?
      1. Follow up: Would you say this impact the same or different than it was at the start of the pandemic?
7. During the interview you also mentioned… (here you could ask about something specific that parent shared that you find important to follow up, it could be familial factors or social factors). Is this still the same or have you seen any change?
8. Can you tell me about your current work situation?
   1. Follow up: has your work situation changed in the last 6 months?
   2. All: Do you feel that your work situation influences your family’s routines? Or do your family’s routines influence your work situation?
      1. Follow up (if examples needed): food preparation, mealtimes, physical activity, children’s activities
   3. All: Compared to 6 months ago, do you feel that your work situation is more or less stressful, or about the same?
9. Is there anything else that has changed over the last 6 months due to the Coronavirus situation that you think has affected your family?
10. *(This question is only for those for whom you don’t have a recent measurement taken or are planning to measure the child within 2 months of the interview* One final question. To collect statistics on all the children enrolled in the study, we would like to know your child’s most recent weight and height. Have you measured your child’s weight and height recently, that is during the last 2 months?)
    1. If yes: in what context (at pediatrician’s office, elsewhere?)
    2. If no: could you visit us for measurement at the university/clinic?
    3. If no, could you measure your child yourself and provide us the results? We can call you again tomorrow, if that would suit you.

**Examples of follow-up questions during the interview:**

- Could you develop that…
- Can you explain that more in detail…
- What do you mean when you say…
- That sounds interesting, tell me more about that!
- You mentioned something about… can you tell me more about that?
- In what way?
- How do you handle this?
- Can you give me an example?
- Have I understood you correctly if you mean that…
- I would like to go back to what you said about…
- What did it mean to you?
- Can you describe more about that…
